# Language-Driven Cross-Attention for Visible–Infrared Image Fusion Using CLIP

**DOI:** 10.3390/s25165083

**Published:** 2025-08-15

**Authors:** Xue Wang, Jiatong Wu, Pengfei Zhang, Zhongjun Yu

**Affiliations:** Aerospace Information Research Institute, Chinese Academy of Sciences, Beijing 100094, China; wangxue@aircas.ac.cn (X.W.); yuzj@ucas.ac.cn (Z.Y.)

**Keywords:** language, cross attention, clip

## Abstract

Language-guided multimodal fusion, which integrates information from both visible and infrared images, has shown strong performance in image fusion tasks. In low-light or complex environments, a single modality often fails to fully capture scene features, whereas fused images enable robots to obtain multidimensional scene understanding for navigation, localization, and environmental perception. This capability is particularly important in applications such as autonomous driving, intelligent surveillance, and search-and-rescue operations, where accurate recognition and efficient decision-making are critical. To enhance the effectiveness of multimodal fusion, we propose a text-guided infrared and visible image fusion network. The framework consists of two key components: an image fusion branch, which employs a cross-domain attention mechanism to merge multimodal features, and a text-guided module, which leverages the CLIP model to extract semantic cues from image descriptions containing visible content. These semantic parameters are then used to guide the feature modulation process during fusion. By integrating visual and linguistic information, our framework is capable of generating high-quality color-fused images that not only enhance visual detail but also enrich semantic understanding. On benchmark datasets, our method achieves strong quantitative performance: SF = 2.1381, Qab/f = 0.6329, MI = 14.2305, SD = 0.8527, VIF = 45.1842 on LLVIP, and SF = 1.3149, Qab/f = 0.5863, MI = 13.9676, SD = 94.7203, VIF = 0.7746 on TNO. These results highlight the robustness and scalability of our model, making it a promising solution for real-world multimodal perception applications.

## 1. Introduction

The core of multimodal fusion lies in acquiring complementary information from multiple data sources, thereby compensating for the incomplete data provided by a single modality and achieving a more comprehensive and enriched scene representation [1]. Infrared and visible light image fusion is a highly representative task in this domain [2,3]. Infrared imaging relies on detecting thermal radiation, maintaining high robustness in poorly lit environments and highlighting thermal targets with strong contrast [4]; however, it often lacks sufficient texture details [5,6]. In contrast, visible light images provide abundant structural and textural information but can suffer significant detail loss under low-light conditions or occlusion [7]. By leveraging the complementarity of these two modalities, one can retain the salient thermal information while integrating the clear textural details from visible light images, thus providing a more accurate and comprehensive representation of the original scene. The fused images typically exhibit both prominent target contrast and rich textural detail, offering superior visual perception for human observers and supplying more reliable input for subsequent machine vision tasks [8,9,10].

Language-guided multimodal fusion, which integrates visible and infrared images with linguistic descriptions, has shown great potential in enhancing image fusion performance, especially under low-light and adverse weather conditions. Visible images often carry rich color and texture details but tend to suffer from severe information loss in poorly lit or harsh environments. In contrast, infrared images maintain robustness in such conditions but lack sufficient texture and geometric information. To bridge the gap between these modalities, we incorporate semantic cues extracted from textual descriptions. These cues help align and constrain multimodal features more accurately, while also compensating for missing information in either modality. As a result, the fused 2D images exhibit enhanced texture richness and clearer structural representation, leading to improved robustness and accuracy in downstream tasks.

In computer vision applications, tasks such as detection, navigation, localization, and environmental perception are being applied to increasingly diverse scenarios. For example, prior work has explored object detection from Bayer images [11] to extract low-level visual information directly from raw sensor data, as well as underground object detection using ground-penetrating radar (GPR) [12] to enable 3D mapping and target recognition in complex subterranean environments. These studies reflect the growing demand for detection technologies under extreme visual conditions and across unconventional modalities. Fused images can provide robots with a more comprehensive understanding of the scene, particularly in low-light or obstacle-dense environments. For example, in autonomous driving, the integration of visible and infrared features with semantic guidance enables easier detection of roads and obstacles even in extreme environments such as nighttime or tunnels, thereby enhancing driving safety. Similarly, in rescue missions, semantically enriched fusion results allow robots to maintain accurate perception and navigation in smoke-filled or low-visibility settings, enabling efficient search and rescue operations. Overall, language-guided fusion enhances a system’s adaptability and stability in complex, real-world environments.

To address these challenges, we propose a visible-infrared fusion framework based on vision-language models. This framework employs a cross-domain attention mechanism to extract complementary features across modalities and integrates a language-guided module that embeds semantic information into the fusion process. The resulting fused images not only preserve salient thermal targets from infrared input but also retain rich texture and structural details from visible images, offering more reliable inputs for subsequent computer vision tasks.

**Figure 1:**
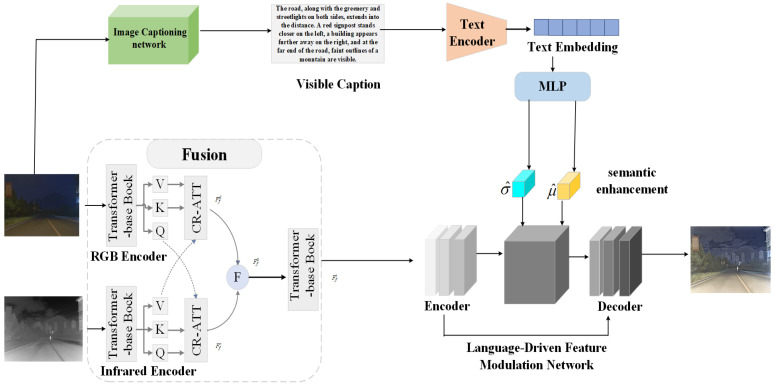
Overview of the framework. For visible and infrared images, individual features are extracted, and cross-attention mechanisms are used to fuse the features from both modalities. Subsequently, a text description corresponding to the visible image is generated using an image-to-text description network. The textual description is encoded through the CLIP text encoder, and a multi-layer perceptron (MLP) is employed to predict semantic information and fusion parameters, effectively guiding the image fusion process.

Due to the lack of explicit supervision from real fused images, we introduce a language-guided fusion process into our framework. By generating textual descriptions for visible-light images, we are able to capture more comprehensive scene details and structural information, while also injecting these high-level semantic cues into the subsequent fusion stages. In the language-driven fusion module, we adopt a network called visible captioning, which takes visible-light images as input and outputs corresponding textual descriptions of the scene. Under the guidance of these descriptions, the fusion process dynamically adjusts the weights and priorities of features according to the scene context, thereby producing fused images that are more semantically aligned with real-world environments. Overall, our contributions are summarized as follows: 1. We propose a language-driven, vision-language-based infrared and visible image fusion framework, significantly enhancing fusion performance and interpretability. 2. We introduce a cross-domain attention mechanism to effectively integrate multimodal features from visible and infrared images, leveraging the complementary advantages of both modalities. 3. We design a semantic interaction guidance module, which extracts textual semantic information from content descriptions of visible-light images to guide the generation of high-quality fused images from multimodal features. As illustrated in Figure 1, our framework outlines the entire processing flow and the interplay among its constituent modules. 4. We achieved the best overall performance across multiple metrics on both the TNO and LLVIP datasets.

## 2. Related Work

### 2.1. Conventional Image Fusion Methods:

Sparse representation theory models image signals as a linear combination of as few “atoms” or transformation primitives as possible, derived from an overcomplete dictionary [13]. This approach is especially powerful in image fusion because it captures data-driven representations by learning a complete dictionary that can adapt to diverse image features, allowing for efficient feature extraction and representation [14]. In practice, sparse representation methods excel at emphasizing intrinsic structures (e.g., salient edges, key textures) while suppressing irrelevant details. Advances in dictionary learning—such as structured sparsity, adaptive dictionary construction, and online dictionary learning—have further strengthened their ability to represent complex image patterns, widening the scope of possible applications in fusion tasks. Such methods have proven highly effective in scenarios that demand high-precision feature selection, including medical imaging diagnostics and remote sensing fusion, where minimal loss of critical detail is paramount. Multi-scale transformation techniques break down the original image into sub-bands or sub-images at multiple scales, mirroring the hierarchical processing observed in the human visual system. By decomposing images into various levels of detail, these methods help preserve fine-grained information and important structures during the fusion process [2,15]. Representative approaches include nonlinear methods, pixel-level weighted averaging, estimation-based procedures, and color composite fusion [16,17,18,19,20]. Through effectively merging complementary information from the source images, multi-scale methods enhance the clarity, sharpness, and informativeness of the final fused outputs. Recent innovations, such as wavelet and curvelet transforms, further boost the ability to capture multi-resolution features. This multi-level understanding of the scene makes multi-scale transformation techniques especially suitable for application fields like medical diagnostics, night-vision enhancement, and surveillance, where preserving both large-scale structures and subtle details is critical. Subspace representation approaches project high-dimensional image features onto a low-dimensional subspace, thereby efficiently capturing essential structures in the input images while reducing computational overhead and memory consumption [21]. Widely adopted techniques include Principal Component Analysis (PCA) [22,23], Independent Component Analysis (ICA) [24,25], and Non-Negative Matrix Factorization (NMF) [26,27]. These methods typically focus on extracting the most salient features from the source images and discarding redundant or noise-related information, thus facilitating an informative and efficient fusion process. Subspace-based approaches have demonstrated considerable success in diverse applications, such as medical imaging (where dimensionality reduction can reveal latent anatomical structures), remote sensing (where large-scale datasets necessitate efficient processing), and multi-modal data integration (where merging data from various sensors requires robust feature-level transformations). Saliency detection seeks to emulate human visual behavior by identifying the most conspicuous regions or objects in an image. In computer vision and pattern recognition, such models play a pivotal role in tasks like object detection, image segmentation, and scene understanding [25,27,28]. When applied to image fusion, saliency-based methods generally involve computing spatial weights and extracting salient objects so that essential visual information is retained in the fused image [29,30,31,32,33]. Recent strides in deep learning-based saliency detection—leveraging convolutional neural networks (CNNs) or Transformer-based architectures—have led to heightened accuracy and robustness, making these techniques more adaptable to complex and dynamic scenarios. Notably, in infrared-visible fusion, saliency models help maintain the most critical infrared targets while preserving the contextual detail from visible imagery, resulting in output images that effectively integrate both modalities. Collectively, these classical approaches—sparse representation, multi-scale transformation, subspace representation, and saliency detection—form an essential foundation for image fusion. They complement data-driven deep learning methods by offering strong theoretical grounding and well-defined transform domains. By leveraging the strengths of each technique and integrating them with emerging deep architectures, researchers continue to push the boundaries of multimodal fusion, leading to more accurate, robust, and application-specific solutions.

### 2.2. Deep Learning-Based Image Fusion Methods

Convolutional neural networks (CNNs) have been widely explored for image fusion tasks, yielding a series of high-performing methods. One representative approach is PMGI [34], which employs a lightweight architecture to preserve gradient and intensity information effectively, enabling quick and robust fusion in practical applications. To further enhance texture detail retention, SDNet [35] introduces an optimization objective grounded in texture richness. This method incorporates an adaptive decision module to balance gradient preservation and texture representation, resulting in more comprehensive fusion outputs even in complex scenarios. Additionally, STDFusionNet [3] addresses the challenge of identifying critical regions by incorporating a salient target mask as spatial guidance, thus reinforcing the fusion of key objects from multiple modalities. Refs. [36,37] extract textual descriptions from depth priors to assist depth estimation. Ref. [38] extracts visible and non-visible features using a diffusion model and guides image fusion based on the textual descriptions of depth. A number of studies have also examined the impact of illumination on fusion quality. For instance, refs. [39,40] systematically analyze how varying lighting conditions influence the performance of image fusion algorithms, thereby offering insights into potential optimizations. Their findings underscore the importance of designing fusion strategies that can adapt to diverse lighting situations for more consistent and reliable results. Ref. [41] investigates image fusion for images with different focal lengths. In recent years, the combined use of CNN and Transformer architectures for multimodal image fusion has demonstrated considerable promise. By leveraging the self-attention mechanism inherent in Transformers, methods such as SwinFusion [42] and SwinFuse [43] capitalize on long-range dependencies to exploit the complementary nature of features across different modalities. These hybrid approaches not only improve fusion quality in various multimodal tasks but also exhibit strong generalization capabilities, extending the applicability of image fusion techniques to a broader range of real-world scenarios.

### 2.3. Language–Vision Models

As deep network architectures continue to evolve at a rapid pace and large-scale datasets become increasingly available, language–vision models are gaining prominence in the field of generative modeling. Among these, CLIP [44] stands as a foundational work. It employs two neural network encoders to process text and images separately, achieving semantic alignment through contrastive learning. Leveraging unsupervised training and extensive data, CLIP exhibits robust zero-shot recognition capabilities and highly efficient feature extraction for both text and images, thereby laying a solid groundwork for subsequent text-driven image generation and processing methods. Building upon CLIP, Style-CLIP [45] has become a highly influential application. It integrates StyleGAN’s [46] powerful image generation capabilities with textual prompts, enabling users to edit images interactively via natural language—a flexible and user-friendly approach to personalized image editing. Beyond GAN-based models, text-conditioned diffusion models have also garnered attention for their superior generation quality and flexibility. For instance, DiffusionCLIP [47] leverages diffusion models in conjunction with CLIP’s semantic understanding to perform text-driven image processing, while Stable Diffusion [48] further incorporates text encoders and attention mechanisms to achieve outstanding text-guided image generation. These methods commonly support interactive multimodal fusion and fine-grained control, greatly expanding the possibilities for image creation and editing. Addressing the limitations of existing methods in handling degraded or complex scenes, TextIF [49] proposes a text-guided image fusion framework that allows users to generate customized fused images via interactive text prompts. Meanwhile, ref. [50] employs CLIP to map text into a multimodal embedding space, representing fusion targets and image features through relationships among embedding vectors and introducing a language-driven loss function to optimize fusion performance. Despite notable progress in related technologies, most existing methods rely on relatively simple textual information, making it difficult to capture the full complexity of multimodal fusion tasks. To address this issue, we introduce an enhanced strategy: extracting more comprehensive and diverse fusion features from both visible and infrared images, and generating object-level textual descriptions from visible images to enrich textual information at a finer granularity. Our experiments demonstrate that this approach not only significantly improves the accuracy and reliability of fused images but also better accommodates a wide range of complex application scenarios, thereby creating a broader space for continued progress in multimodal image synthesis.

## 3. Method

RGB images typically capture abundant texture, color, and shape information, providing a detailed representation of the scene for both human and computer vision systems. However, in extreme environments (e.g., nighttime, smoke, heavy fog) or in scenarios with severe occlusion, RGB images often suffer substantial loss of visible information. By contrast, non-visible light (e.g., infrared or thermal imaging) can effectively detect thermal radiation or other spectral signals under these conditions, thereby strongly complementing the limitations of traditional RGB images. By leveraging a cross-attention mechanism to fuse features from these two distinct modalities, the system can fully exploit their complementary advantages at the feature level, achieving more comprehensive and robust perception of the environment and targets.

### 3.1. Image Encoder

In this module, both the RGB image and the non-visible light image (e.g., infrared) are used as inputs to the encoder. To extract crucial information from both visible and non-visible modalities, we employ a Transformer/Restormer-based network structure [51] as the primary feature extractor. This process can be formalized as follows:

(1) $$F_{rgb}=F_{r}^{I}\left(I_{ir}\right),\;F_{niv}=F_{niv}^{I}\left(I_{niv}\right),$$

where $I_{ir}\in R^{H\times W\times 3}$ is the three-channel RGB image, and $I_{niv}\in R^{H\times W\times 1}$ denotes the single-channel non-visible light image. *H* and *W* represent the image height and width, respectively. $F_{r}^{I}$ and $F_{niv}^{I}$ are the encoders for RGB and non-visible light data, respectively.

Within each encoder, the Transformer/Restormer architecture leverages self-attention, feed-forward networks, and multi-scale feature pyramids to perform feature extraction at multiple resolutions. This design not only captures global semantic context at larger scales but also preserves local texture details at finer scales, laying a solid foundation for subsequent cross-modal information interaction.

### 3.2. Cross-Modality Feature Fusion

Non-visible light provides extended coverage or captures target information in spectral bands beyond human vision, such as temperature distribution, shape contours, and localized heat sources. Meanwhile, RGB images offer color, texture, and high-resolution details. To fully exploit their complementary advantages, we adopt a cross-attention mechanism (CR-ATT) in the cross-modality fusion layer, aiming to strengthen the interaction and information flow between non-visible light and RGB features. The resulting fused features enable deeper collaboration across different modalities, yielding a more discriminative and robust multi-modal representation. First, we project the RGB features $F_{rgb}$ and non-visible light features $F_{niv}$ into {Q,K,V} components:

(2) $$\left\{Q_{r},K_{r},V_{r}\right\}=F_{qkv}^{r}\left(F_{rgb}\right)$$

(3) $$\left\{Q_{d},K_{d},V_{d}\right\}=F_{qkv}^{d}\left(F_{niv}\right)$$

where $F_{qkv}^{r}$ and $F_{qkv}^{d}$ are linear transformations that generate queries, keys, and values for RGB and non-visible light features, respectively. Next, to encourage further spatial interaction between the two modalities, we exchange their query vectors:

(4) $$F_{f}^{d}=\mathrm{softmax}\left(\frac{Q_{r}K_{d}^{T}}{d_{k}}\right)V_{d}$$

(5) $$F_{f}^{r}=\mathrm{softmax}\left(\frac{Q_{d}K_{r}^{T}}{d_{k}}\right)V_{r}$$

where $d_{k}$ is a scaling factor to maintain numerical stability. Here, $F_{f}^{d}$ represents the features obtained by combining RGB queries with non-visible keys and values, while $F_{f}^{r}$ is derived from combining non-visible queries with RGB keys and values. Through this cross-attention, each modality can “focus” on the most informative regions or channels in the other modality, thereby strengthening the representation of specific targets or regions of interest.

Finally, we concatenate $F_{f}^{d}$ and $F_{f}^{r}$ along the channel dimension:

(6) $$F_{f}^{0}=\mathrm{Concat}\left(F_{f}^{d},F_{f}^{r}\right),$$

(7) $$F_{f}^{i}=F_{\mathrm{tran}}\left(F_{f}^{0}\right),$$

$F_{tran}$ refers to a Transformer layer that performs further feature extraction on the directly concatenated multimodal features. Thus yielding the fused multi-modal feature representation. This representation integrates spatial and geometric information from non-visible light with the color and texture details from RGB images, proving beneficial for downstream tasks such as image fusion, object detection, or semantic segmentation.

By using cross-attention to fuse RGB and non-visible light features, the system can more effectively aggregate information from multiple modalities, demonstrating superior performance in complex and dynamically changing real-world environments. In low-light or smoke-filled settings, infrared or thermal images offset the limitations of RGB-based observation; under normal lighting conditions, RGB features provide more intuitive visual details. By leveraging these two types of information simultaneously, we not only gain higher accuracy in target recognition and localization but also substantially improve the system’s adaptability in applications such as autonomous driving, surveillance, medical image analysis, and nighttime navigation.

### 3.3. Language-Driven Image Fusion

In the image fusion process, deep learning techniques typically rely on multiple loss functions to constrain the model. However, the absence of real fused images for direct supervision makes it challenging to effectively regulate the fusion output via loss functions. Moreover, many problems cannot be explicitly modeled, thereby limiting the model’s overall performance. Motivated by the idea that text descriptions generated from images can provide valuable visual cues, we propose a language-guided fusion method. Specifically, we adopt ExpansionNet v2 from [52], which takes an RGB image as input and outputs a textual description of the image content. Given a pair of RGB images $I_{ir}$ and niv images $I_{niv}$, the RGB image is first processed by the image captioning network to generate the corresponding text description. These text descriptions are then concatenated and fed into the frozen text encoder of CLIP, resulting in text embedding features that encapsulate information about the RGB image. To utilize these text embedding features for guiding image fusion, we extract a set of semantic parameters from the text embeddings. These parameters contain high-level guidance information such as object attributes and spatial relationships. We then employ a Multilayer Perceptron (MLP) to learn these relationships and further map the textual semantic information ($\hat{\sigma }$ and v) for feature $F_{f}^{i}$ scaling and bias control, respectively. The process is formulated as follows:

(8) $${\hat{F}}_{f}^{i}=\left(1+\hat{\sigma }\right)⊙F_{f}^{i}+\hat{\mu },$$

where ⊙ denotes the Hadamard product (element-wise multiplication).

In this formulation, the semantic parameters interact with the fused features through a feature modulation mechanism, specifically via scale and bias adjustments. This allows the model to flexibly refine multimodal representations from a semantic perspective, injecting richer contextual information into the fusion process, enhancing feature expressiveness, and achieving effective alignment of multimodal features within the semantic space. Moreover, we introduce residual connections within the language-driven feature modulation network to alleviate gradient vanishing issues during deep network training, reduce the difficulty of model fitting, and enhance the stability of the fusion process. Under semantic guidance, the model can adaptively assign appropriate weights and priorities to different features, making the fusion results more aligned with the semantic requirements of specific scenes. The final fused image exhibits clear semantic structures and strong hierarchical representation while preserving critical information from both target and background, thus laying a solid perceptual foundation for downstream tasks such as image understanding, detection, and recognition.

### 3.4. Loss Function

Loss Function for the Fusion Process: To ensure that the fused image retains both structural details and intensity characteristics from the source modalities, we introduce two complementary loss functions: a multi-channel gradient loss and a multi-channel intensity loss. To preserve fine texture and edge information, we employ the multi-channel gradient loss $L_{MCG}$, which encourages gradient consistency between the fused image and the most salient gradients from either the visible or infrared input. It is defined as follows:

(9) $$\begin{array}{c} L_{MCG}=\frac{1}{HW}\sum_{i=1}^{3}{∥\nabla I_{f}^{i}-\max\left(\nabla |I_{ir}^{i}\left|,\nabla \right|I_{niv}^{i}|\right)∥}_{1} \end{array}$$

where ∇ denotes the gradient operator, and $I_{f}^{i}$ represents the *i*-th channel (i.e., red, green, or blue) of the fused image $I_{f}$. Similarly, $I_{ir}^{i}$ denotes the *i*-th channel of the visible image $I_{ir}$. To ensure that the fused image maintains overall brightness and structural coherence with the source inputs, we also apply the multi-channel intensity loss $L_{MCI}$, formulated as follows:

(10) $$\begin{array}{c} L_{MCI}=\frac{1}{HW}\sum_{i=1}^{3}{∥I_{f}^{i}-\max\left(I_{ir}^{i},I_{niv}^{i}\right)∥}_{1} \end{array}$$

The final loss function used to train the fusion network is a weighted combination of these two components:

(11) $$L_{f}=L_{MCG}+L_{MCI}$$

This joint loss encourages the fused image to retain both sharp edge information and meaningful intensity patterns from the source modalities, facilitating high-quality color fusion.

## 4. Experiment

### 4.1. Implementation Details and Datasets

Datasets: We conducted comprehensive experiments on three widely used public datasets for visible-infrared fusion: LLVIP [53], and TNO [54]. These datasets encompass a diverse range of scenes, object types, lighting conditions, and thermal distributions, providing a solid benchmark to evaluate the generalization ability and robustness of our proposed language-guided fusion method. Specifically, the LLVIP dataset contains paired RGB and infrared images collected in indoor and outdoor environments under different illumination settings (daytime, nighttime, and low-light), while the TNO dataset focuses on military and surveillance scenarios with strong thermal contrast. These diverse characteristics help validate the practical applicability of our method across multiple domains.

Metrics: Five statistical metrics are adopted for quantitative evaluation, namely Mutual Information (MI) [55], Visual Information Fidelity (VIF) [56], Spatial Frequency (SF) [57], Q_abf_ [58], and Standard Deviation (SD). Collectively, they offer a comprehensive assessment of the fused image from multiple perspectives:

- MI (Mutual Information): Evaluates the degree to which information from both source images is preserved and integrated in the fused result, indicating how well the fusion process combines complementary details.
- VIF (Visual Information Fidelity): Measures the fidelity of the fused image relative to the source images, focusing on how accurately essential visual content is conveyed.
- SF (Spatial Frequency): Examines the spatial frequency components within the fused data, reflecting the level of detail and sharpness retained.
- Q_abf_: Quantifies the edge information contributed by each source image, offering insight into how effectively structural details are preserved in the final result.
- SD (Standard Deviation): Assesses the overall contrast of the fused image, highlighting its dynamic range and distinguishing ability between different intensity levels.

These metrics jointly capture various dimensions of image quality—ranging from information integration and detail clarity to structural fidelity and contrast—thus providing a well-rounded evaluation of fusion performance.

### 4.2. Analysis of Fusion Results with Textual Guidance

**Figure 2:**
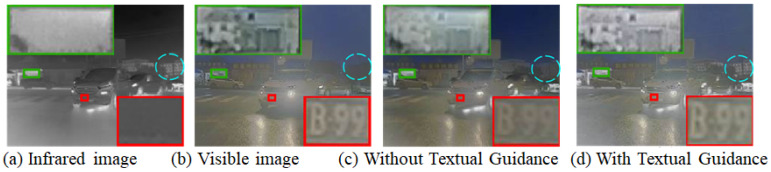
Comparison of fusion results with and without textual guidance. (**a**) Infrared image; (**b**) Visible image; (**c**) Fused result without textual guidance; (**d**) Fused result with textual guidance. The red, green, and blue boxes highlight key semantic regions including license plate details, building structures, and traffic signs, respectively.

As illustrated in Figure 2, subfigures (a) and (b) show the original infrared and visible images, respectively. Subfigures (c) and (d) present the fused results without and with textual guidance. A comparative analysis reveals several notable improvements brought by the textual guidance module:

Enhanced detail clarity: In subfigure (d), the license plate region (highlighted by the red box) exhibits significantly sharper edges and higher character recognizability (e.g., “B92”) than in subfigure (c). This suggests that textual guidance enhances the model’s semantic attention to critical targets such as license plates, leading to more precise feature preservation in the fusion output.

Improved texture retention: The architectural textures enclosed in the green box are better preserved in the text-guided result. Compared to (c), subfigure (d) demonstrates clearer structural boundaries and reduced blurring, indicating that textual semantics help maintain structural integrity and detail fidelity.

Salient semantic target enhancement: The traffic sign in the blue circle is more distinct and easier to recognize in (d) than in (c), with sharper edges and improved contrast. This highlights the effectiveness of language-guided fusion in reinforcing visually and semantically important elements.

These improvements stem from the textual guidance mechanism, which extracts high-level semantic cues (e.g., “license plate”, “building”, “traffic sign”) from image descriptions and encodes them into attention-aware parameters. These semantic priors are then used to guide the fusion process, enabling the network to focus on meaningful regions while suppressing redundant or ambiguous background information. Consequently, the final fused image achieves better semantic consistency, structural completeness, and perceptual quality.

### 4.3. Comparison of Visible and Infrared Image Fusion

**Figure 3:**
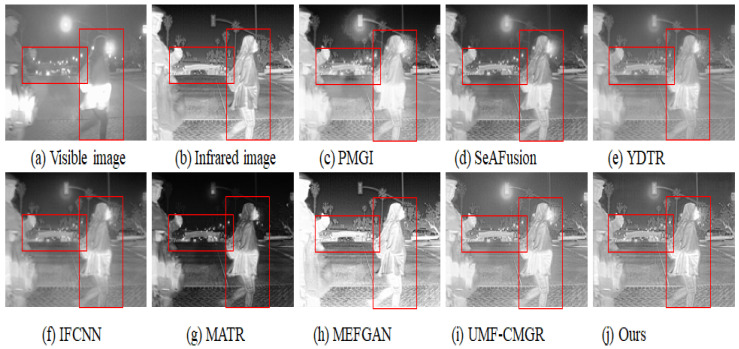
Infrared and visible image fusion experiment on “pedestrian crossing” images from the TNO dataset. PMGI [59], SeAFusion [9], YDTR [60], IFCNN [61], MATR [62], MEFGAN [63], UMF-CMGR [64], ours.

**Figure 4:**
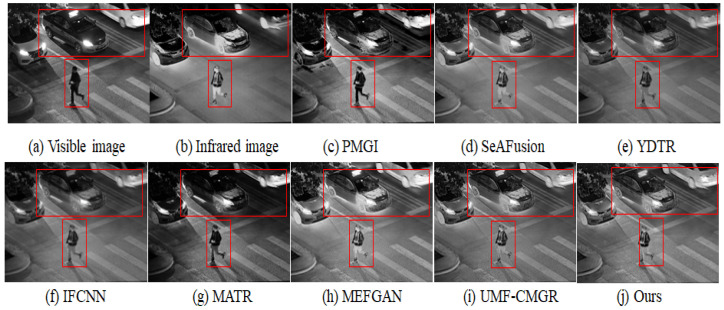
Infrared and visible image fusion experiment on “nighttime vehicles and pedestrians” images from the LLVIP dataset. PMGI [59], SeAFusion [9], YDTR [60], IFCNN [61], MATR [62], MEFGAN [63], UMF-CMGR [64], ours.

**Figure 5:**
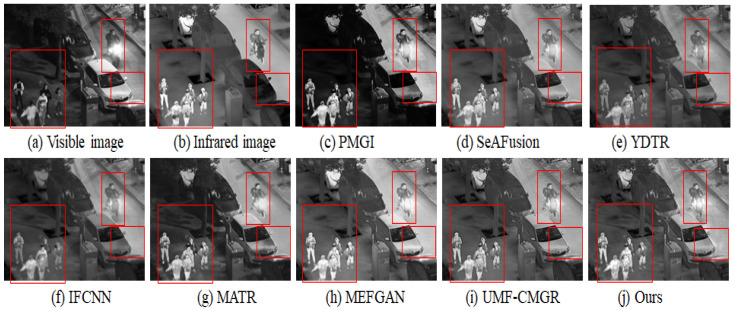
Infrared and visible image fusion experiment on “multiple pedestrians” images from the LLVIP dataset. PMGI [59], SeAFusion [9], YDTR [60], IFCNN [61], MATR [62], MEFGAN [63], UMF-CMGR [64], ours.

In Figure 3, Figure 4 and Figure 5, we present three sets of fusion results of visible and infrared images for the scenarios “pedestrian crossing,” “nighttime vehicles and pedestrians,” and “multiple pedestrians,” respectively. We use red boxes to indicate the areas in the fused images where differences most likely occur among the various methods. Compared to the other models, our method demonstrates notable advantages in the following aspects:

#### 4.3.1. Saliency and Detail Preservation of Pedestrians

Infrared images generally highlight pedestrians’ thermal radiation but appear somewhat blurry in terms of texture and contours. Visible images, on the other hand, contain abundant detail yet may lose key information under insufficient illumination. From the three sets of figures, it is evident that in processing pedestrian areas, other models either diminish the high-contrast features in the infrared image, causing pedestrians to be less prominent, or fail to adequately preserve the texture from the visible image, resulting in blurring or distortion in pedestrian clothing or outlines. In contrast, our method effectively combines the intense highlights from infrared with the fine textures from visible images, ensuring that pedestrians in the final fused image remain both clear and highly recognizable.

#### 4.3.2. Clarity of Background and Noise Suppression

In the right-side red box of Figure 3, as well as in the background regions of Figure 4 and Figure 5, there is often substantial noise or variations in lighting. Many of the compared models still retain noticeable noise after fusion or cannot simultaneously present the structured information from the visible image and the overall brightness from the infrared image. In our method’s cross-modal feature interaction, local and global information are dynamically balanced, allowing the background to maintain appropriate brightness and texture details while effectively reducing noise, thereby avoiding the issue of the image being “too dark” or “too bright.”

#### 4.3.3. Distant Targets and Scene Structure

In the top red box of Figure 5 (the “pedestrians”) and the distant background in Figure 4 (vehicles), some far-off targets or scene structures are visible. In the fusion results of other models, such distant objects often appear blurred or lost. Through deeper interactions of multi-modal features, our method performs more precise texture extraction and contrast enhancement for distant areas, thus more completely retaining the shapes and structural information of these targets.

#### 4.3.4. Overall Visual Consistency and Layered Appearance

Infrared images may show overexposure in regions with high temperatures or reflective surfaces, while visible images could lose details at night or in shadowed areas. By adaptively balancing exposure and shadowing between these two modalities, our method achieves a better equilibrium between detail preservation and overall visual quality, resulting in a stronger sense of depth and layering. Whether it is pedestrians, vehicles, or background buildings, the final fused image achieves a relatively natural visual effect.

#### 4.3.5. Overall Performance Assessment

Taken together, the results from these three sets of experiments indicate that our method not only preserves the salient targets from infrared images and the detailed information from visible images but also effectively controls noise and prevents exposure imbalance. The visual and quantitative evaluations both demonstrate strong stability and superior performance. As shown in Table 1, our algorithm achieves the highest score in 4 of the 5 evaluation metrics and ranks third in the remaining one; Table 2 similarly confirms that we maintain leading performance on a large-scale, high-resolution dataset. Through both visual inspection and numerical assessment, it can be concluded that our method exhibits significant overall advantages in visible-infrared image fusion.

**Figure 6:**
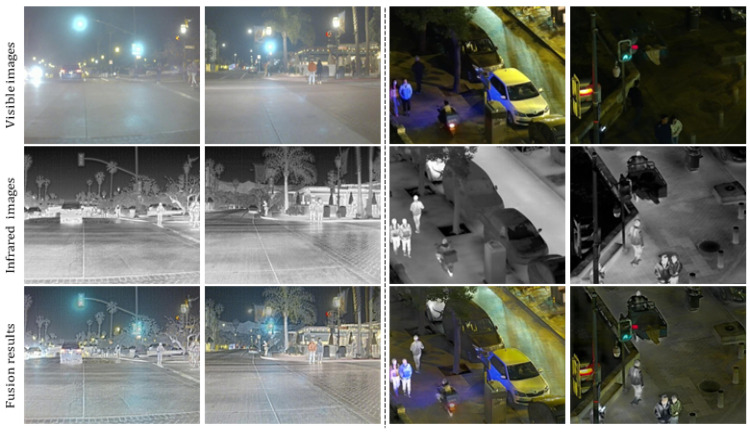
Infrared and visible image fusion experiment on LLVIP dataset.

Figure 6 presents visible light images, infrared images, and the fusion results under nighttime scenes involving multiple pedestrians, vehicles, and complex backgrounds. Based on the comparison, the advantages of our fusion method are evident: Visible images contain rich detail information such as road textures, traffic signs, and clothing of individuals. However, under low-light conditions, they suffer from poor illumination, making target recognition more difficult. Infrared images clearly highlight thermal features of pedestrians and vehicles, offering strong target detection capability. However, they lack structural and texture details, resulting in weak overall scene representation. Fusion results effectively combine the advantages of both modalities. They retain detailed information from the visible images—subtext descriptions containing RGB information serve as interpretable prior guidance for image fusion results such as road structures, object contours, and background textures—while also enhancing the prominent thermal targets captured by the infrared images, making key objects like pedestrians and vehicles more distinct and clear. Specifically, in the fusion images, pedestrian clothing and facial contours are not only thermally highlighted but also show enhanced texture details. The appearance of vehicles and surrounding objects is more natural and realistic. Notably, in the third and fourth columns, the fusion results significantly improve the visibility of multiple targets in nighttime scenes, demonstrating strong environmental adaptability and enhanced visual clarity. This indicates that our proposed method performs robustly and is practically valuable in low-light and complex environments.

### 4.4. Impact of Caption Variability on Fusion Performance

To evaluate the robustness of our method to different forms of textual input, we employed multiple image captioning models to generate diverse textual descriptions from the same visible-light images. These descriptions vary in wording and phrasing but all broadly reflect the semantic content of the input image. We then conducted fusion and evaluated the results using standard quantitative metrics. As shown in Table 3, the performance across different textual inputs remains highly consistent, indicating that as long as the generated descriptions are semantically aligned with the image content, their impact on fusion quality is minimal. This demonstrates the flexibility and generalizability of our language-guided fusion framework.

### 4.5. Dataset-Specific Differences in Structural Metrics

We observe a notable disparity in structural and perceptual metrics—namely Spatial Frequency (SF), Standard Deviation (SD), and Visual Information Fidelity (VIF)—between the LLVIP and TNO datasets. As shown in Table 1 and Table 2, all compared methods achieve consistently higher SF, SD, and VIF scores on LLVIP, whereas the corresponding values on TNO are significantly lower. This phenomenon can be attributed to several key differences between the two datasets:

(1) Resolution and texture density of input images. LLVIP is a high-resolution dataset primarily composed of near-field, human-centered scenes captured under various illumination conditions. It contains rich texture details, well-defined object boundaries, and fine structural edges—especially in the RGB modality. As SF and SD are sensitive to spatial variations and intensity contrast, the richer visual content in LLVIP naturally leads to higher metric values. In contrast, the TNO dataset consists mainly of medium-to-long-range surveillance imagery, where both infrared and visible images have relatively lower resolution and fewer fine details. The infrared images in particular exhibit smoother transitions and blurred contours, resulting in lower structural fidelity in the fused output and subsequently lower SF and SD values.

(2) Thermal contrast and dynamic range differences. TNO infrared images typically exhibit strong thermal contrast, with hot targets (e.g., humans or vehicles) appearing bright against dark backgrounds. This limited dynamic range may hinder the model’s ability to preserve fine visual information, thereby reducing the VIF score. In LLVIP, the infrared and visible modalities are better aligned in terms of brightness distribution, allowing for more balanced fusion and clearer preservation of both modalities’ information, which contributes to consistently higher VIF scores.

(3) Scene conditions and target applications. LLVIP is designed for pedestrian perception tasks in nighttime or low-light environments, where detailed semantic and structural information is critical. As such, the fusion results emphasize edge clarity and texture preservation, leading to elevated values in SF, SD, and VIF. TNO, on the other hand, is oriented toward long-range surveillance and defense-related scenarios, where the focus is on enhancing thermal target saliency rather than preserving high-frequency texture or visual fidelity. This results in a shift of emphasis in the fusion process, explaining the lower structural metric values observed.

### 4.6. Ablation Study

Feature Fusion Module: This module achieves efficient multimodal feature fusion by integrating Transformer-based feature extraction with a cross-attention mechanism. Specifically, the Transformer feature extraction component captures long-range dependencies and global contextual information within multimodal data, thereby extracting deeper and more discriminative features from RGB and infrared images. The cross-attention mechanism then dynamically computes correlation weights between the RGB and infrared features, effectively highlighting complementary key information and suppressing redundant or noisy signals. This fusion strategy fully exploits the complementary strengths of the two modalities in both structure and detail, resulting in fused images that are clearer and richer in visual content. To further validate the effectiveness of this module, we removed it while keeping other components intact and compared the results with the evaluation metrics listed in Rows 1 and 2 of Table 4. After removing the feature fusion module, all metrics declined to varying degrees, indicating that it plays a critical role in extracting multimodal features and enhancing the fused image quality.

Language-Driven Fusion Module: In this module, text descriptions containing image information serve as interpretable prior guidance for image fusion. We design an MLP to predict fusion parameters that dynamically adjust the weights and priorities of the RGB and infrared features during the fusion process. To test its effectiveness, we fixed the MLP-predicted parameters to 1 and 0, thereby removing the influence of language on the image fusion. Comparing Rows 2 and 3 of Table 4, we observe a significant performance drop without language guidance, indicating that the RGB priors embedded in the text descriptions effectively improve fusion quality. By integrating these text-based constraints, the fused images achieve higher fidelity and clarity, while also offering enhanced interpretability for multimodal fusion.

### 4.7. Limitations

Although our qualitative analysis demonstrates advantages in terms of target clarity, background reconstruction, and visual consistency, it still has certain limitations. First, qualitative evaluation is inherently subjective, and the results may be influenced by individual viewer preferences or perceptual differences, making it difficult to ensure complete objectivity. Second, while the selected image examples are representative, they may not fully cover all possible scene variations, such as extreme weather conditions or complex background occlusions. Third, qualitative analysis alone cannot comprehensively reflect the effectiveness of the fused images in downstream tasks such as object detection or depth estimation, which limits the depth of assessment regarding the method’s practical applicability. In future work, we plan to incorporate more task-driven evaluation methods, combining both subjective and objective metrics to further validate the robustness and generalizability of our model in real-world applications.

## 5. Conclusions

In this paper, we propose a language-guided framework for fusing infrared and visible light images, which integrates both visual features and semantic cues to enhance fusion quality. The overall architecture consists of two main components: a cross-modal fusion branch, which leverages cross-domain attention to effectively combine visible and infrared modalities, and a language-driven module, which introduces high-level semantic guidance into the fusion process. To obtain the semantic descriptions, we employ an off-the-shelf image captioning model to extract textual descriptions from visible light images. These descriptions are then encoded using the CLIP text encoder to generate semantic representations, which guide the feature modulation and enhance the fusion outcome. By aligning visual content with language-derived semantics, our method enables the generation of high-quality, color-enhanced fusion results. This approach not only improves interpretability but also demonstrates strong potential for practical applications in low-light surveillance, medical diagnostics, and remote sensing scenarios.

## Figures and Tables

**Table 1:** Quantitative evaluation results on the TNO dataset.

Method	SF	Qab/f	MI	SD	VIF
DeepFuse [65]	8.3500	0.3847	13.2205	66.8872	0.5752
DenseFuse [66]	9.3238	0.4735	13.7053	81.7283	0.6875
RFN-Nest [59]	5.8457	0.3292	13.4547	67.8765	0.5404
PMGI [59]	8.7195	0.3787	13.7376	69.2364	0.6904
U2Fusion [67]	11.0368	0.3934	13.4453	66.5035	0.7680
IFCNN [61]	11.8590	0.4962	13.2909	73.7053	0.6090
FusionGAN [68]	8.0476	0.2682	13.0817	61.6339	0.4928
MEFGAN [63]	7.8481	0.2076	13.9454	43.7332	0.7330
SeAFusion [9]	11.9355	0.4908	14.0663	93.3851	0.8919
YDTR [60]	3.2567	0.1410	12.3865	56.0668	0.2792
MATR [62]	5.3632	0.2723	13.0705	78.0720	0.3920
UMF-CMGR [64]	8.2388	0.3671	12.6301	60.7236	0.3934
**Ours**	11.3149	0.5863	13.9676	94.7203	0.7746

**Table 2:** Quantitative evaluation results on the LLVIP dataset.

Method	SF	Qab/f	MI	SD	VIF
DeepFuse [65]	12.4175	0.4620	14.0444	0.4586	38.3328
DenseFuse [66]	12.5900	0.4700	14.0723	0.4669	38.7011
RFN-Nest [59]	10.6825	0.3844	14.1284	0.4658	39.7194
PMGI [59]	12.0997	0.3951	14.0737	0.4487	37.9572
U2Fusion [67]	17.2889	0.4985	13.4141	0.4917	37.4284
IFCNN [61]	21.7698	0.6092	14.4835	0.6762	44.0938
FusionGAN [68]	9.2062	0.0600	12.8981	0.1141	26.9133
MEFGAN [63]	15.1905	0.3644	13.9575	0.8720	59.7947
SeAFusion [9]	20.9194	0.6181	14.9016	0.8392	51.8096
YDTR [60]	7.0755	0.1961	13.3858	0.3365	33.1625
MATR [62]	13.5066	0.4282	11.9989	0.4575	34.1515
UMF-CMGR [64]	13.4481	0.3707	13.4037	0.3841	35.1731
**Ours**	22.1381	0.6329	14.2305	0.8527	45.1842

**Table 3:** Quantitative results under different image captioning models on the TNO dataset.

Method	SF	Qab/f	MI	SD	VIF
GLaMM [69]	8.3500	0.3847	13.2205	66.8872	0.5752
Perceive [70]	9.3238	0.4735	13.7053	81.7283	0.6875
ExpansionNet v2 [52]	11.3149	0.5863	13.9676	94.7203	0.7746

**Table 4:** Ablation study of our methods on the TNo: Cro: Cross Attention. Lan: Language-Driven Fusion Module.

Cross	Lan	SF	Qab/f	MI	SD	VIF
		7.3240	0.4432	8.6232	51.4228	0.6238
✓		9.4612	0.5196	12.4140	72.4543	0.7426
✓	✓	11.3149	0.5863	13.9676	94.7203	0.7746

## Data Availability

The original contributions presented in this study are included in the article. Further inquiries can be directed to the corresponding author.
